# Characterization of the barrier conferred by type I interferon on the early steps of HIV-1 infection in primary cells

**DOI:** 10.1186/1742-4690-8-S2-P26

**Published:** 2011-10-03

**Authors:** Caroline Goujon, Hélène Bauby, Fransje A Koning, Michael H Malim

**Affiliations:** 1 King's College London, Infectious Diseases Department, London, SE1 9RT, UK

## Background

Type I interferon (IFN) induces blocks to various steps of viral life cycles. It has been known for a long time that treatment of certain cell types with IFN confers a potent block to both the early and late steps of HIV-1 replication. However, the early block has remained poorly characterized so far.

## Material and methods

We have investigated in detail the effect of type I IFN on HIV-1 infection in cell types relevant to natural infection, including primary CD4+ T cells, monocyte-derived macrophages, as well as several monocytic and T cell lines.

## Results

We have found that IFN treatment of macrophages, THP-1 cells, and to a lesser extent CD4+ T cells, has a profound effect on the outcome of HIV-1 infection, whereas the effect is much less pronounced in U937 cells and minimal in various T cell lines. We have also shown that IFN exerts potent anti-viral effects against a broad range of retroviruses, including diverse HIV-1 strains (IIIB, BK132, NL4-3, YU-2, Ba-L), HIV-1 derived lentiviral vectors (LVs), SIVmac and FIV LVs, as well as B-MLV. Using a BlaM-Vpr entry assay, we have observed that viral entry is not affected by IFN treatment. More specifically, the block directly correlates with a strong decrease in the accumulation of viral cDNAs.

As APOBEC3 proteins are IFN regulated, we have asked whether they have the capacity to edit reverse transcripts generated by incoming wild type HIV-1 viruses in macrophages. Using single genome sequencing, we have shown that viral cDNAs from IFN treated macrophages bear a low but statistically significant amount of G-to-A mutations that are acquired post-entry. Based on the sites that are subjected to editing, we predict that APOBEC3 proteins beyond APOBEC3G play a role in this process.

## Conclusions

Taken together, our results indicate that one or several effectors of the type I IFN response are able to block viral DNA accumulation of distant retroviruses in primary macrophages and CD4+T cells. In addition, we have shown that the classic hallmark of APOBEC3-mediated editing is observed on viral cDNAs recovered from IFN treated macrophages. Given the low mutational frequency noted in these experiments, it seems unlikely that the editing shown here is an essential effector of the potent IFN induced antiviral effect. Instead it is more likely to provide an additional mechanism to augment other sources of HIV-1 sequence diversification.

